# The Agr-Like Quorum-Sensing System Is Important for *Clostridium perfringens* Type A Strain ATCC 3624 To Cause Gas Gangrene in a Mouse Model

**DOI:** 10.1128/mSphere.00500-20

**Published:** 2020-06-17

**Authors:** Mauricio A. Navarro, Jihong Li, Juliann Beingesser, Bruce A. McClane, Francisco A. Uzal

**Affiliations:** a California Animal Health and Food Safety Laboratory System, School of Veterinary Medicine, University of California, Davis, San Bernardino, California, USA; b Department of Microbiology and Molecular Genetics, University of Pittsburgh School of Medicine, Pittsburgh, Pennsylvania, USA; University of Kentucky

**Keywords:** *Clostridium perfringens*, Agr-like quorum-sensing system, gas gangrene

## Abstract

Clostridium perfringens type A is involved in gas gangrene in humans and animals. Following a traumatic injury, rapid bacterial proliferation and exotoxin production result in severe myonecrosis. C. perfringens alpha toxin (CPA) and perfringolysin (PFO) are the main virulence factors responsible for the disease. Recent *in vitro* studies have identified an Agr-like quorum-sensing (QS) system in C. perfringens that regulates the production of both toxins. The system is composed of an AgrB membrane transporter and an AgrD peptide that interacts with a two-component regulatory system in response to fluctuations in the cell population density. In addition, a synthetic peptide named 6-R has been shown to interfere with this signaling mechanism, affecting the function of the Agr-like QS system *in vitro*. In the present study, C. perfringens type A strain ATCC 3624 and an isogenic *agrB*-null mutant were tested in a mouse model of gas gangrene. When mice were intramuscularly challenged with 10^6^ CFU of wild-type ATCC 3624, severe myonecrosis and leukocyte aggregation occurred by 4 h. Similar numbers of an *agrB*-null mutant strain produced significantly less severe changes in the skeletal muscle of challenged mice. Complementation of the mutant to regain *agrB* expression restored virulence to wild-type levels. The burdens of all three C. perfringens strains in infected muscle were similar. In addition, animals injected intramuscularly with wild-type ATCC 3624 coincubated with the 6-R peptide developed less severe microscopic changes. This study provides the first *in vivo* evidence that the Agr-like QS system is important for C. perfringens type A-mediated gas gangrene.

**IMPORTANCE** Clostridium perfringens type A strains produce toxins that are responsible for clostridial myonecrosis, also known as gas gangrene. Toxin production is regulated by an Agr-like quorum-sensing (QS) system that responds to changes in cell population density. In this study, we investigated the importance of this QS system in a mouse model of gas gangrene. Mice challenged with a C. perfringens strain with a nonfunctional regulatory system developed less severe changes in the injected skeletal muscle compared to animals receiving the wild-type strain. In addition, a synthetic peptide was able to decrease the effects of the QS in this disease model. These studies provide new understanding of the pathogenesis of gas gangrene and identified a potential therapeutic target to prevent the disease.

## Abstract (precis)

Clostridium perfringens type A strains produce toxins that are responsible for clostridial myonecrosis, also known as gas gangrene. Toxin production is regulated by an Agr-like quorum-sensing (QS) system that responds to changes in cell population density. In this study, we investigated the importance of this QS system in a mouse model of gas gangrene. Mice challenged with a C. perfringens strain with a nonfunctional regulatory system developed less severe changes in the injected skeletal muscle compared to animals receiving the wild-type strain. In addition, a synthetic peptide was able to decrease the effects of the QS in this disease model. These studies provide new understanding of the pathogenesis of gas gangrene and identified a potential therapeutic target to prevent the disease.

## INTRODUCTION

Clostridium perfringens is a Gram-positive, anaerobic, spore-forming bacterium that is responsible for a number of human and animal diseases due to the production of several toxins (1–3). Toxin production patterns vary among individual strains. This variability permits a classification system that assigns C. perfringens isolates to one of seven types (A to G) based upon the presence of the alpha (CPA), beta (CPB), epsilon (ETX), iota (ITX), enterotoxin (CPE), and necrotic enteritis B-like (NetB) toxin genes (4).

C. perfringens type A is the main cause of clostridial myonecrosis (gas gangrene) in humans and animals. The disease commonly starts with the infection of soft tissue, particularly muscle, by C. perfringens spores or vegetative cells as a result of a traumatic injury (5). Gas gangrene is clinically characterized by pain, fever, local edema, gas production, and necrosis of skeletal muscle, usually progressing to toxemia, shock, sepsis, and often death (6). The main virulence factor of C. perfringens for producing gas gangrene is CPA (7), a toxin with phospholipase C and sphingomyelinase activities that is encoded by the *cpa* (*plc*) gene (8). In addition, the pore-forming toxin perfringolysin O (PFO), encoded by the *pfoA* gene, acts synergistically with CPA during the pathogenesis of gas gangrene (9). CPA and PFO alter the extravasation of inflammatory cells, decreasing the infiltration of such cells to the site of infection (6, 10, 11). Both toxins have also been shown to induce upregulation of adhesion molecules on the surface of inflammatory cells, which would promote intravascular cell aggregation, followed by vascular occlusion (11–15).

Bacterial pathogens often regulate their virulence gene expression in response to environmental signals (16). This regulation commonly involves two, sometimes cross-talking, regulatory systems named two-component regulatory systems (TCRS) and quorum-sensing (QS) systems (16, 17). QS systems control gene expression in response to bacterial population density through the production and detection of autoinducing peptides (AIPs), a group of small extracellular signaling molecules that sometimes bind to and activate the membrane sensor component of a TCRS (18–20).

The accessory gene regulator (Agr) system in Staphylococcus aureus is a prototype regulatory system involving both TCRS and QS systems. It consists of four cotranscribed genes: *agrB*, *agrD*, *agrC*, and *agrA* (21). The *agrD* gene encodes the AIP, which is processed to the active form by the AgrB transporter and then secreted extracellularly. Once a sufficient concentration of the AIP accumulates in the extracellular environment to trigger activation of the AIP-binding AgrC membrane sensor, the AgrC/AgrA TCRS then regulates gene expression (21).

Similar Agr-like regulatory systems are present in other Gram-positive pathogens, including C. perfringens. The C. perfringens genome carries an Agr-like operon encoding both an AgrD peptide and an AgrB membrane transporter (22, 23). This Agr-like operon is highly conserved among C. perfringens strains but, at least for predicted AIPs, is quite different from the Agr-like QS systems present in other pathogenic clostridia (24). However, unlike S. aureus, the Agr-like QS locus of C. perfringens does not appear to directly encode a TCS. Instead, it has been proposed that the VirR/VirS TCRS often performs this function (25) based upon observations that expression of several C. perfringens toxin genes, including those encoding CPA, PFO, CPB and NetB, are controlled by both the Agr-like QS system and the VirR/VirS TCRS (25–30).

Another difference between the Agr systems of S. aureus and C. perfringens concerns their AIPs. The S. aureus AIP, which varies among strains, is a 7- to 9-amino-acid peptide containing a five-member thiolactone ring with a short amino acid tail. In contrast, the AIP in the C. perfringens Agr-like QS system is likely a tailless five-member thiolactone ring whose amino acid sequence differs from the thiolactone ring in the S. aureus AIP (31). Interfering with AIP signaling can affect Agr-like QS regulation, e.g., a synthetic peptide named 6-R, which is a six-membered thiolactone ring containing the natural five amino acids of the C. perfringens AIP plus an adjacent amino acid, reduces production of CPB by some C. perfringens type B and type C strains (31).

Since *agrB*- or *agrD*-null mutants of C. perfringens are impaired for the *in vitro* production of CPA and PFO (25, 30) and both of these toxins are important for type A strains to cause gas gangrene, the present study directly investigated whether the Agr-like QS system regulates the virulence of C. perfringens gas gangrene in a mouse model. For this purpose, wild-type C. perfringens type A strain ATCC 3624, an *agrB*-null mutant and a complemented strain were used. In addition, the possible inhibitory effects of the 6-R peptide on C. perfringens virulence in this gas gangrene model was tested to further evaluate if the Agr-like QS system is important for gas gangrene.

## RESULTS

### Construction and genotypic characterization of an ATCC 3624 *agrB*-null mutant and complementing strain.

**FIG 1:**
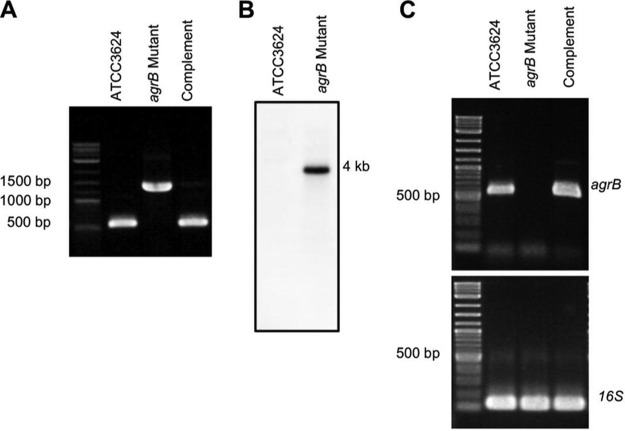
Intron-based mutagenesis to create an *agrB*-null mutant of C. perfringens type A wild-type ATCC 3624. (A) PCR confirmation of the construction of an *agrB*-null mutant of wild-type ATCC 3624. Using DNA isolated from wild-type ATCC 3624, a PCR assay amplified a 536-bp product using internal primers, but a larger 1.5-kb product, consistent with the insertion of a 0.9-kb intron, using template DNA isolated from the *agrB* mutant. A similar PCR analysis confirmed the presence of the 536-bp product in the complementing strain. (B) Southern blot hybridization of an intron-specific probe with DNA from wild-type ATCC 3624. DNA from each strain was isolated and electrophoresed on a 1% agarose gel prior to blotting and hybridization with an intron-specific probe. The size of the DNA fragment, in kilobases (kb), is shown at the right. (C) RT-PCR evaluation of *agrB* expression. Reverse transcription of extracted RNA from each strain, followed by PCR, was performed. Expression of the *agrB* gene was demonstrated for the wild-type parent and the complementing strain, but no *agrB* transcription was detectable for the ATCC 3624 *agrB*-null mutant. All three ATCC 3624-related strains expressed similar levels of 16S RNA.

The present study constructed an *agrB*-null mutant of type A strain ATCC 3624 in order to begin evaluating whether the Agr-like quorum-sensin*g* system regulates the ability of type A strains to produce toxins and cause gas gangrene in a mouse model. For this purpose, the *Clostridium*-modiﬁed TargeTron-mediated insertional mutagenesis method (32) was used to construct an ATCC 3624 *agrB*-null mutant, as demonstrated by PCR using primers that flank the intron insertion site and are speciﬁc for internal *agrB* open reading frame (ORF) sequences (Fig. 1A). With template DNA from wild-type ATCC 3624, these internal PCR primers speciﬁcally ampliﬁed a PCR product of 536 bp. However, the same primers ampliﬁed a PCR product of about 1.5 kb using DNA from the putative *agrB* mutant, which is consistent with the insertion of a 900-bp intron into the *agrB* ORF. The intron delivery plasmid was then cured by subculturing for 10 days in the absence of antibiotic selection.

To confirm that only a single intron insertion was present in the putative mutant, DNA was isolated from this strain and subjected to Southern blot analysis using an intron-speciﬁc probe (Fig. 1B). The intron-speciﬁc probe did not hybridize with wild-type DNA on this Southern blot, as expected. In contrast, this Southern blot experiment revealed that DNA from the *agrB*-null mutant strain contained a single intron insertion.

A complementing strain was then prepared using a plasmid where the *agr* operon was cloned into the C. perfringens/Escherichia coli shuttle plasmid pJIR750 (30). After this plasmid was transformed into the ATCC 3624 *agrB*-null mutant by electroporation, PCR confirmed the presence of the wild-type *agrB* ORF in the complementing strain. Note that, while Fig. 1A shows the presence of the intron-disrupted *agrB* gene in the mutant used to prepare the complementing strain, this larger PCR product was not amplified from the complementing strain. This phenomenon has been observed many times previously with complementation of mutants created by intron-mediated insertional mutagenesis (33–36). It is due to the primers amplifying products from both the wild-type and intron-disrupted *agrB* genes in the complementing strain but because of its much smaller size, the PCR product from the wild-type *agrB* is created more rapidly and greatly increases in relative abundance after each PCR round. An RT-PCR assay was then used (Fig. 1C) to assess *agrB* expression by 5-h TY broth cultures of wild type, the *agrB*-null mutant and the complementing strain since an AgrB antibody is not available. This reverse transcription-PCR (RT-PCR) analysis conﬁrmed that wild-type ATCC 3624 expresses *agrB* transcripts. However, no *agrB* transcription was detectable for the mutant. The complementing strain showed restored *agrB* transcription with expression levels similar to those of the wild-type strain. For all three ATCC 3624-related strains, the 16S RNA expression levels were similar.

### Comparison of growth rates and toxin production by wild-type ATCC 3624 versus an ATCC 3624 *agrB*-null mutant or complementing strain in TY medium.

**FIG 2:**
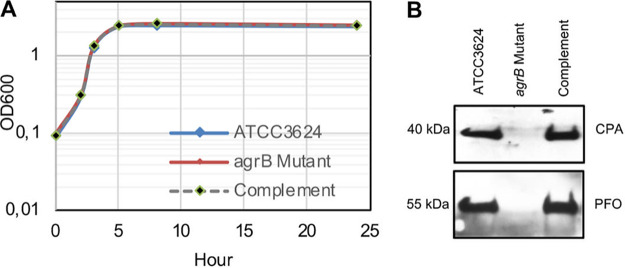
Comparison of growth or CPA and PFO production by wild-type ATCC 3624, the *agrB* mutant, and the complementing strain. (A) Cultures of each strain were grown to 24 h at 37°C in TY medium. At the designated time points, the OD_600_ of each culture was determined. Representative results of three repetitions for each strain are shown. (B) CPA and PFO Western blotting performed with the supernatant from the overnight culture of each strain grown overnight at 37°C in TY medium. The molecular mass is indicated on the left. Representative Western blots for four repetitions are shown.

When growth curve analyses were performed (Fig. 2A), wild-type ATCC 3624, its *agrB*-null mutant, and the complementing strain all grew similarly in TY medium at 37°C.

Since type A strains commonly produce CPA and PFO, Western blot studies were performed to compare CPA and PFO production by ATCC 3624, the *agrB*-null mutant and the complementing strain after overnight (about 16 h) growth in TY medium at 37°C. The results (Fig. 2B) confirmed CPA and PFO production by wild-type ATCC 3624. However, there was no detectable CPA or PFO production by the *agrB*-null mutant, even after overnight culture. The *agrB* complementing strain made similar amounts of CPA and PFO as the wild-type parent. These results indicate that, as reported previously for type A strain 13 (25, 30), CPA and PFO production are strongly upregulated by the *agr* locus in type A strain ATCC 3624.

### Virulence of wild-type *C. perfringens* type A strain ATCC 3624 and its derivatives in a mouse model of gas gangrene.

The present study next tested the pathogenicity of C. perfringens type A strain ATCC 3624 and its derivatives in a mouse model of clostridial myonecrosis. For this analysis, ∼10^6^ CFU of wild-type ATCC 3624, the *agrB*-null mutant and complementing strain were each inoculated intramuscularly into the left hind leg of eight male or female BALB/c mice (weighing 20 to 25 g) per group. A fourth group of eight mice was injected intramuscularly with sterile Dulbecco phosphate-buffered saline (DPBS; control). After 4 h of incubation, the mice were euthanized and examined for gross pathology, and samples were collected for microscopic evaluation and C. perfringens immunohistochemistry (IHC).

**FIG 3:**
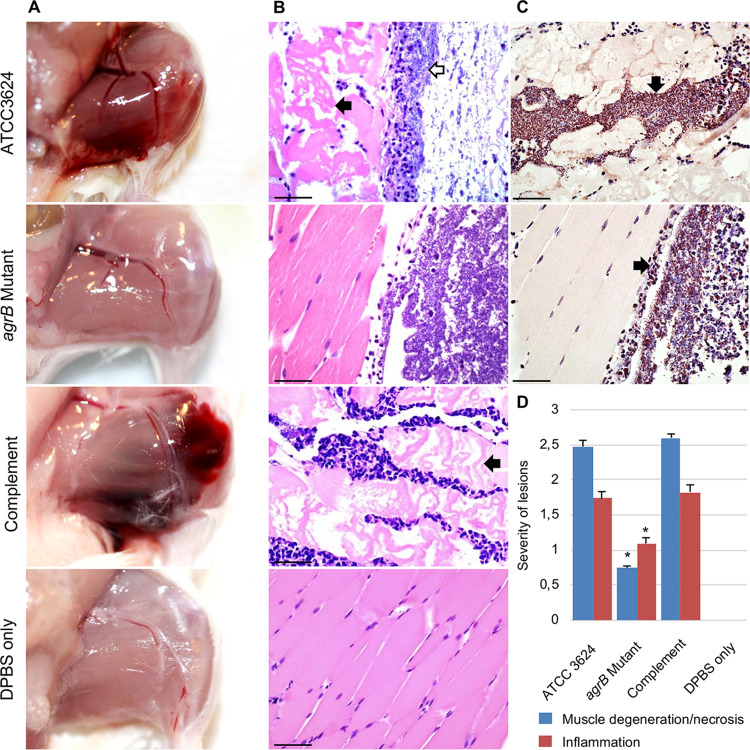
Virulence effects of *agrB* gene inactivation in a mouse model of C. perfringens type A-induced gas gangrene. The hind limbs of mice were injected with ∼10^6^ CFU of wild-type ATCC 3624, *agrB* mutant, complementing strain, or DPBS only and then incubated for 4 h. (A) Macroscopic pathology. Changes in skeletal muscle of mice injected with wild-type ATCC 3624 and the complementing strain are characterized by swelling, edema, and hemorrhage. No gross lesions were observed in mice receiving the *agrB* mutant or DPBS buffer. (B) Microscopic pathology of the corresponding treatments. Note the myriad rod-shaped bacteria (open arrow) in association with myonecrosis (solid arrow) in a mouse challenged with wild-type ATCC 3624 or the complementing strain but the absence of lesions in the skeletal muscle of mice receiving the *agrB* mutant or DPBS. (C) C. perfringens immunohistochemistry on the skeletal muscle of a mouse challenged with wild-type ATCC 3624 or the *agrB* mutant showing large numbers of brown, short rods (black arrows). Panels A to C show representative results for eight mice receiving each treatment. (D) Histological score of skeletal muscles treated with the indicated inocula for 4 h. Error bars show the standard errors of the means. *, *P* < 0.05. Scale bar, 25 μm.

Grossly, wild-type ATCC 3624 and the complementing strain induced severe changes in challenged mice; these changes were characterized by swelling and dark-red discoloration (hemorrhage) of the affected skeletal muscle. In contrast, a similar challenge with the *agrB*-null mutant induced no gross changes in mouse skeletal muscle (Fig. 3A). The negative control, i.e., an injection of DPBS buffer, also failed to induce any gross changes in skeletal muscle.

Microscopically, wild-type ATCC 3624 induced severe histological changes, characterized by muscle degeneration, necrosis, and inflammation with myriad intralesional rods. Leukostasis was prominent within multiple blood vessels (Fig. 3B). Similar challenge with the *agrB* mutant produced muscle degeneration, necrosis and inflammation with myriad intralesional rods that were significantly less severe than those observed in mice inoculated with the wild-type strain. The severity of these changes reverted to wild-type levels in animals receiving the complementing strain. Mice receiving DPBS only showed no changes in any of the evaluated parameters (Fig. 3B and D). Association of C. perfringens with the microscopic lesions in mice inoculated with wild-type ATCC 3624, the *agrB* mutant (Fig. 3C), or complementing strain was confirmed by IHC using an indirect immunoperoxidase technique.

### Recovery of viable *C. perfringens* from challenged skeletal muscle.

**FIG 4:**
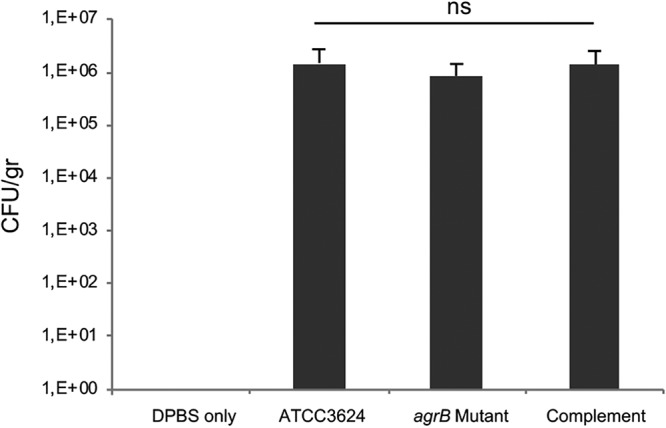
Recovery of viable C. perfringens from challenged tissues. After 4 h of incubation, samples of skeletal muscle from mice challenged with ∼10^6^ CFU of wild-type ATCC 3624, the *agrB* mutant, the complementing strain, and DPBS only were aseptically collected and plated on TSC agar for CFU calculations of C. perfringens. Error bars show standard errors of the means. ns, not significant. Each bar represents the mean results using samples from eight mice.

The IHC results indicated that the amounts of total (live and dead) C. perfringens in muscle was similar after challenge with ATCC 3624 or its derivatives (Fig. 3C). To assess whether the numbers of viable C. perfringens present in these tissues were similar, skeletal muscle was aseptically collected from all mice and then plated on C. perfringens selective agar to process for CFU calculations. Similar numbers (∼10^6^) of viable C. perfringens were recovered 4 h after challenge from all mice receiving wild-type ATCC 3624, its *agrB*-null mutant, or the complementing strain (Fig. 4). Fifteen randomly selected colonies were screened by PCR for the presence of the *cpa* gene to confirm their identity as C. perfringens. All colonies tested positive (data not shown). No C. perfringens were isolated from mice injected with DPBS buffer alone (controls).

### Effects of the 6-R synthetic signaling peptide on *in vitro* CPA or PFO production by ATCC 3624.

**FIG 5:**
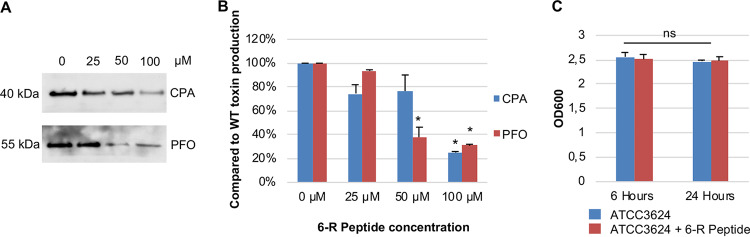
Effects of the 6-R synthetic signaling peptide on *in vitro* CPA or PFO production by ATCC 3624. (A) Representative CPA and PFO Western blots performed with supernatants from overnight cultures of the wild-type ATCC 3624, coincubated with three different concentrations of the 6-R peptide in TY medium grown overnight at 37°C. The molecular mass is indicated on the left. (B) CPA and PFO production levels in the presence of the 6-R peptide (100 μM) compared to the wild-type levels using ImageJ densitometric analyses of three separate Western blots. (C) Cultures of the ATCC 3624 strain with or without 6-R peptide (100 μM) were grown to 24 h at 37°C in TY medium. At the designated time points, the OD_600_ of each culture was determined. Representative results of three repetitions for each strain are shown. Error bars show the standard errors of the means. *, *P* < 0.05. The results shown in panel A are representative of three Western blot experiments for each toxin, and panels B and C show the mean of three repetitions.

The 6-R peptide is a thiolactone ring consisting of the likely natural AIP of C. perfringens plus an additional amino acid that inhibits, by an unidentified mechanism, the Agr-like QS system of several type B and type C wild-type C. perfringens strains, resulting in reduced CPB toxin production by those type B and C strains (31). Therefore, the present study evaluated whether the 6-R peptide can also inhibit CPA or PFO production by type A strain ATCC 3624. For this analysis, three different concentrations of the 6-R peptide were added to TY broth inoculated with wild-type ATCC 3624, followed by CPA or PFO Western blot analysis of CPA or PFO levels in 5-h culture supernatants of those cultures. The results showed (Fig. 5A) that, at a 100 μM concentration, the 6-R peptide caused an ∼75% reduction in both CPA and PFO production. For PFO production, even a 50 μM concentration of the 6-R peptide had significant inhibitory effects (Fig. 5B). This reduction in toxin production was not due to the 6-R peptide affecting bacterial growth (Fig. 5C).

### Effects of the 6-R synthetic signaling peptide on *C. perfringens* type A-induced gas gangrene *in vivo*.

**FIG 6:**
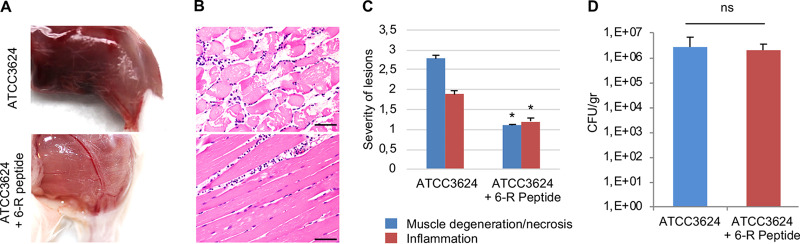
Inhibitory effects of the 6-R peptide on the virulence of ATCC 3624 in a mouse model of C. perfringens type A-induced gas gangrene. The hind limbs of mice were injected with ∼10^6^ CFU of wild-type ATCC 3624 with or without the 6-R peptide (100 μM) and incubated for 4 h. (A) Macroscopic pathology of mice injected with the corresponding treatment. (B) Microscopic pathology of mice injected with the corresponding treatment. Note the severe myonecrosis in a mouse receiving the ATCC 3624 strain only. Only a few inflammatory cells (neutrophils) are seen between the muscle fibers in a mouse receiving the 6-R peptide (100 μM). (C) Histological scores of skeletal muscles treated with the indicated inocula for 4 h. (D) Comparison of growth yields by C. perfringens type A ATCC 3624 strain coincubated with or without the 6-R peptide (100 μM) recovered from challenged skeletal muscle of mice. Error bars show standard errors of the means. *, *P* < 0.05; ns, not significant. The data in the panels are representative of eight mice each. Scale bar, 50 μm.

Since the 6-R peptide was able to reduce *in vitro* production of both CPA and PFO, the present study used this inhibitory peptide to further evaluate involvement of the Agr-like QS system in gas gangrene by testing whether 6-R affects virulence in the mouse model of gas gangrene induced by C. perfringens type A infection. For this experiment, ∼10^6^ CFU of wild-type ATCC 3624 was incubated for 4 h with a 100 μM concentration of the 6-R peptide in dimethyl sulfoxide (DMSO) or an equal concentration of DMSO (control) and then injected into the leg muscles of mice. After 4 h, muscle degeneration/necrosis and inflammation were significantly less severe in animals receiving the 6-R peptide (100 μM) than in mice receiving wild-type ATCC 3624 alone (Fig. 6A to C). Similar numbers (∼10^6^) of viable C. perfringens were recovered 4 h after challenge from all mice receiving wild-type ATCC 3624 or this strain plus 6-R peptide (100 μM) (Fig. 6D). Fifteen randomly selected colonies were screened by PCR for the presence of the *cpa* gene to confirm their identity as C. perfringens. All colonies tested positive (data not shown). No bacteria were isolated from mice injected with DPBS alone (controls).

## DISCUSSION

The present study establishes an important role for the Agr-like QS system in the pathogenesis of gas gangrene induced by C. perfringens type A in a mouse model. Similar QS communication systems are present across many pathogenic firmicute species (37) where they commonly function as regulators for concerted population behaviors, including biofilm formation, sporulation, and virulence (38–40). It has been hypothesized that the coordinated production of extracellular virulence factors when a sufficient population density is reached can induce considerable damage to the host, reducing, at the same time, the use of metabolic resources (29).

In appropriate C. perfringens types, the Agr-like QS system regulates *in vitro* production of most extracellular toxins (25, 28–30, 35, 41). Involvement of the Agr-like QS in C. perfringens infections originating in the intestine was previously demonstrated for type C (28) and type G (29) strains. In the type C study, an *agrB*-null mutant of type C strain CN3685 showed reduced enteropathogenicity in a rabbit intestinal loop model and less lethality in a mouse enterotoxemia model; both effects were reversible by complementation. Furthermore, attenuation of this *agrB* mutant was attributed to reduced production of CPB (28). Similarly, the virulence of an *agrB*-null mutant of type G strain CP1 was attenuated in a chicken necrotic enteritis model *in vivo* and pathogenicity was restored by complementation (29). Even though NetB production was not quantified in the referred to *in vivo* study, it is likely that a similar event described for CPB may be responsible for such reduction in pathogenicity in the chicken model since NetB production is controlled by the Agr-like QS and NetB is essential for type G strain-induced chicken necrotic enteritis (29).

Consistent with previous observations (25, 30) using type A strain 13, inactivation of the *agrB* gene in ATCC 3624 also produced a significant *in vitro* decrease in *cpa* and *pfoA* expression at the transcriptional level and production of these protein toxins at the translational level. This reduced toxin expression is likely to be responsible for the significant decrease in the severity of macro- and microscopic lesions induced in the skeletal muscle of mice challenged with the *agrB* mutant since CPA and PFO play central roles in the development of gas gangrene (7, 9). There was a significant reduction in inflammatory cells at the site of injury in mice receiving the ATCC 3624 *agrB*-null mutant, but many cells of this C. perfringens strain were still visible. This reduced presence of inflammatory cells is consistent with previous observations, in which C. perfringens type A strains deficient in CPA or PFO production induced significantly reduced leukocyte aggregation in mice (42).

To test whether the reduced virulence of the *agrB*-null mutant in the gas gangrene model was due to a decreased ability of this mutant to survive and reproduce *in vivo*, skeletal muscle from challenged mice was aseptically collected by the time of euthanasia for C. perfringens counting. The number of viable C. perfringens recovered from muscles 4 h after infection was approximately the same as the challenge dose, indicating that C. perfringens does not significantly grow in this infection model, in agreement with results of another recent study (43). Importantly, no significant differences were observed in the numbers of viable C. perfringens recovered after challenge with the wild-type, mutant, or complemented strains. These results strongly suggest that the reduced virulence of the ATCC 3624 *agrB*-null mutant in the gas gangrene model was not related to differences in its ability to survive *in vivo*.

For ethical reasons this study used a 4-h model based on a pilot study in which this time was sufficient to produce gross and microscopic lesions of gas gangrene, reduce animal suffering and death. This is a modification from previous studies that reported significant clinical sign variations using longer periods models (7).

A previous study showed that, via an unidentified mechanism, a C. perfringens AgrD sequence-based synthetic peptide named 6-R was able to interfere with CPB production by several C. perfringens type B and C strains *in vitro* (31). Why the 6-R peptide blocks or reduces toxin production in only some C. perfringens strains is not understood but it has been speculated that some degree of diversity in C. perfringens AIP receptors may exist (31). The present study determined that the 6-R peptide can also reduce, by ∼75%, the production of CPA and PFO by ATCC 3624 *in vitro*. Cultures of wild-type ATCC 3624 incubated in the presence of a 100 μM concentration of the 6-R peptide also had reduced ability to produce significant lesions in the skeletal muscle of mice. The reduction of both CPA/PFO production *in vitro*, as well as the reduced virulence *in vivo*, was not attributable to the 6-R peptide affecting bacterial growth.

In summary, the present study provides the first evidence that the Agr-like QS system is important for the pathogenesis of gas gangrene in a mouse model of the disease. Given the continuous search for therapies against bacterial pathogens and to avoid the development of antibiotic resistance, interfering with the Agr QS system by using AIP analogues like 6-R may represent a potential candidate as a target to prevent some C. perfringens-mediated diseases, including gas gangrene.

## MATERIALS AND METHODS

### Bacteria, media, and reagents.

C. perfringens type A strain ATCC 3624 was purchased from the American Type Culture Collection (ATCC). The following broth media were used in this study: fluid thioglycolate medium (FTG; Difco Laboratories), TY broth (3% tryptic soy broth [Becton, Dickinson], 1% yeast extract [Becton, Dickinson], and 0.1% sodium thioglycolate [Sigma-Aldrich]), and TGY broth (TY broth supplemented with 2% glucose [Sigma-Aldrich]). The *agrB*-null mutant and complementing strains were screened using brain heart infusion (BHI) agar (Research Products International) plates containing 15 μg ml^−1^ chloramphenicol (Sigma-Aldrich). Tryptose-sulfite-cycloserine (TSC) agar plates made of SFP agar base (Becton, Dickinson) with 0.04% d-cycloserine (Sigma-Aldrich) were used for isolation of C. perfringens from challenged skeletal muscle of mice. All other chemical reagents used in this study were purchased from Fisher Scientific, Sigma-Aldrich, or Bio-Rad.

### Peptide synthesis.

Based upon results of a previous report (31), this study used a synthetic 6-mer (6-R) peptide which consists of the sequence CLWFTH in a thiolactone ring, Synthesis of the 6-R peptide was carried out by the Peptide and Peptoid Synthesis Core Facility Division of the Health Sciences Core Research Facilities (HSCRF) at the University of Pittsburgh. Synthesis was performed using standard FMOC (9-fluorenylmethoxy carbonyl) chemistry cycles on a Liberty CEM microwave synthesizer using Oxyma/DIC [ethyl-(*2Z*)-2-cyano-2-hydroxyiminoacetate/*N*,*N*-diisopropylcarbodiimide] activation in dimethylformamide (DMF). Briefly, FMOC-Thr(tBu), FMOC-His(trt), FMOC-Cys(trt), FMOC-Leu, FMOC-Trp(Boc), and FMOC-Phe were purchased from Peptides International and used for stepwise assembly of the linear sequence on *bis*(2-sulfanylethyl) amino polystyrene (SEA-PS) resin (0.11 mmol g^−1^; Millipore Sigma). Cleavage of the 6-R-*bis*(2-sulfanylethyl) amino intermediate from the SEA solid support was accomplished using a mixture of TFA/TIPS/DMS/H_2_O/thioanisole (90/2.5/2.5/2.5/2.5) for 2 h at room temperature and then precipitated with diethyl ether, followed by three ether washes. The resulting pellet containing the crude 6-R-*bis*(2-sulfanylethyl) amino intermediate was allowed to air dry and then dissolved in 0.2 M sodium phosphate/tris(2-carboxyethyl)phosphine (TCEP) at pH 4.0, layered with N_2_, and incubated overnight at 37°C with gentle stirring. The resulting crude reaction mixture was directly loaded onto a Waters Prep 4000 series chromatography system and purified on a Phenomenex Gemini (21.2 × 250 mm) 10-μm C_18_ column using standard acetonitrile/0.1% TFA gradient conditions. Final analytical determination of peptide purity for the cyclic 6-R-thioester was performed on a Waters Alliance chromatography system using a Phenomenex Gemini (4.6 × 250 mm) 5-μm C_18_ column along with standard acetonitrile/0.1% TFA gradient conditions. Matrix-assisted laser desorption ionization–time-of-flight analysis on an Applied Biosystems Voyager workstation was used for final confirmation of the expected target mass of each peptide. The purity of the final peptide was >95%. The purified synthetic peptide was resuspended in DMSO (Fisher Scientific) at 50 mM for use and then stored in a –80°C freezer for no longer than 2 weeks. In experiments, the final concentrations of 6-R peptide ranged from 25 to 100 μM, as specified.

### Plasmids and primers.

An *agrB* knockout plasmid named pJIR750agrBNi was constructed to prepare an ATCC 3624 *agrB*-null mutant using *Clostridium*-modified group II TargeTron Technology (32). The intron on this plasmid was sense orientation targeted to insert into the *agrB* ORF between nucleotides 342 and 343. The primers used for intron targeting the *agrB* gene were as follows: agrB-342|343s-IBS, 5′-AAAAAAGCTTATAATTATCCTTAGTGTTCATTGGAGTGCGCCCAGATAGGGTG-3′; agrB-342|343s-EBS1d, 5′-CAGATTGTACAAATGTGGTGATAACAGATAAGTCATTGGAATTAACTTACCTTTCTTTGT-3′; and agrB-342|343s-EBS2, 5′-TGAACGCAAGTTTCTAATTTCGATTAACACTCGATAGAGGAAAGTGTCT-3′. The 350-bp intron PCR product was then inserted into pJIR750ai (32) between the HindIII and BsrGI enzyme (New England Biolabs) sites to construct the pJIR750agrBNi vector. The screening primers used to verify the *agrB*-null mutant were NagrBKOF (5′-TGGAACTTATGCTCTAATACAAACA-3′) and NagrBKOR (5′-AATCTATAGTTTTTAACAATATATTT-3′). The same primer pair was also used for RT-PCR analysis of *agrB* gene expression. 16S RNA was used as a control housekeeping gene for RT-PCR. In this study, all primers were synthesized by Integrated DNA Technologies.

The *agrB* complementation vector (CPJVp3) was constructed as described previously (30). This plasmid was electroporated into the ATCC 3624 *agrB*-null mutant, and transformants were selected on BHI agar containing 15 μg ml^−1^ of chloramphenicol. The mutant and complementing strains were further confirmed and characterized by PCR, RT-PCR, and Southern blot analyses, as described below.

### *C. perfringens* DNA isolation, PCR, and intron Southern blot analyses.

A MasterPure Gram-positive DNA purification kit was used for DNA extraction from all C. perfringens strains according to the manufacturer’s instructions (Epicenter). PCR for the *agrB* gene was performed using the NagrBOF and NagrBKOR primers. For the wild-type strain the PCR product amplified using these primers was 536 bp, while for the *agrB*-null mutant strain the same primer pair amplified a PCR product of about 1,400 bp due to the insertion of a 900-bp intron.

For Southern blotting, aliquots (3 μg each) of wild-type or *agrB*-null mutant DNA samples were first digested overnight with EcoRI at 37°C according to the manufacturer’s instructions (New England Biolabs). The overnight-digested DNA samples were then electrophoresed on a 1% agarose gel, followed by transfer onto a positively charged nylon membrane (Roche) for hybridization with an intron-specific probe (46). The intron-specific probe was prepared using the PCR DIG probe synthesis kit (Roche) and the intron primers (IBS and EBS2). After hybridization, Southern blots were developed using reagents from a DIG DNA labeling and detection kit (Roche), according to the manufacturer’s instructions.

### *C. perfringens* RNA isolation and RT-PCR analyses.

The wild-type parent ATCC 3624 and derivatives (the *agrB*-null mutant or complementing strain) were grown in TY medium for 5 h at 37°C. Cultures were then pelleted and RNA was extracted using saturated phenol and purified by TRIzol and chloroform (Life Technology and Sigma), as previously described (36). After the absence of DNA was confirmed (36) by subjecting samples to PCR without reverse transcriptase, RNA was quantified by measuring the sample absorbance at 260 nm. An aliquot of 1 μl of puriﬁed RNA (100 ng) was then used in a one-step RT-PCR containing 10 μl of 2× *Taq* master mix (New England Biolabs), 4 U of avian myeloblastosis virus reverse transcriptase (Promega), and *agrB* gene primers (described earlier), with ddH_2_O added to reach a 20-μl total volume. Similarly, 16S RNA RT-PCR was performed as a loading control (36). Reaction mixtures were incubated for 45 min at 45°C to allow cDNA synthesis, and then regular PCR cycling was performed using the following conditions: (i) 95°C for 2 min; (ii) 30 cycles of 95°C for 15s, 50°C for 30 s, and 68°C for 30 s; and (iii) a ﬁnal extension of 68°C for 5 min.

### Measurement of *C. perfringens* growth *in vitro*.

For analysis of C. perfringens *in vitro* vegetative growth, a 0.2-ml aliquot from overnight FTG cultures of the wild type, *agrB*-null mutant, or complementing strain was inoculated into 10 ml of TY medium. The cultures were then incubated at 37°C; at various culture times (0, 1, 3, 5, 8, and 24 h) thereafter, a 1-ml aliquot of culture was removed to measure the optical density at 600 nm (OD_600_) by using a Bio-Rad Smart spectrophotometer.

### Western blot analyses of CPA and PFO production.

TY cultures of the wild-type, *agrB* mutant, or complementing strain were adjusted to equal OD_600_s, and 30-μl portions of supernatants from those cultures were then mixed with 5× SDS-PAGE loading buffer and boiled for 5 min. Agr locus controls the expression of a number of secreted proteins (27), so loading equal amounts of protein in these experiments is not applicable for comparing the relative production of PFO or CPA by wild-type, the *agrB* mutant, or complementing strain. Instead, we adjusted the OD_600_ of each culture to equivalence (since Agr does not affect growth) and then loaded the same volume of supernatant from that culture. Portions (30 μl) of each sample were then electrophoresed on a 10% acrylamide SDS gel, and the separated proteins were transferred onto a nitrocellulose membrane. The membrane was blocked with TBS-Tween 20 (0.05% [vol/vol]) and nonfat dry milk (5% [wt/vol]) for 1 h at room temperature, followed by probing with a 1:1,000 dilution of rabbit polyclonal PFO antibody (36) or a 1:250 dilution of mouse monoclonal CPA antibody (36) overnight at 4°C. Finally, bound antibody was detected with a horseradish peroxidase-conjugated secondary anti-rabbit or anti-mouse antibody (Sigma-Aldrich) and the addition of SuperSignal West Pico chemiluminescence substrate (Fisher Scientific).

### Inhibition of toxin production by the 6-R peptide.

Wild-type ATCC 3624 was cultured overnight at 37°C in FTG before reculture in fresh TY broth overnight at 37°C. After that second overnight growth, a 15-μl aliquot of the TY culture was inoculated into 1 ml of TY with or without the specified concentration of 6-R peptide. The culture without 6-R peptide received 2 μl of DMSO as a control since this amount of DMSO was present in cultures receiving the 6-R peptide. After 5 h of culture at 37°C, the supernatants were collected and used for Western blot detection of CPA or PFO production, as described above. ImageJ analysis was performed on three separate Western blots to determine the fold changes of CPA and PFO in the supernatants.

### Mouse model of gas gangrene.

A 40-μl aliquot from overnight FTG cultures of the wild-type, *agrB*-null mutant, or complementing strain was inoculated into 1 ml of TY medium, followed by incubation at 37°C for 5 h. These cultures were then washed in sterile DPBS. An aliquot of 50 μl, equivalent to ∼10^6^ CFU, was injected intramuscularly in the thighs of eight BALB/c mice (20 to 25 g) per group of treatment. Another group of mice was challenged with 50 μl of sterile DPBS (control). The animals were euthanized by cervical dislocation 4 h after infection, and samples of skeletal muscle were collected. All experiments involving mice were reviewed and approved by the University of California, Davis, Institutional Animal Care and Use Committee (protocol 20513).

### Histopathology.

Sections of challenged skeletal muscle were fixed by immersion in 10% buffered formalin (pH 7.2), for 24 to 72 h. Sections (4 μm thick) were then prepared routinely and stained with hematoxylin and eosin. The sections were examined microscopically by a pathologist in a blinded fashion. A semiquantitative score of lesion severity was assigned to each section using an ordinal scale from 0 (no lesions observed) to 4 (most severe). The following scoring system was used, based on examination of 5, 200× microscopic fields: 0, no lesions; 1, changes observed in 0 to 25% of the area examined; 2, changes observed in 25 to 50% of the area examined; 3, changes observed in 50 to 75% of the area examined; and 4, changes observed in 75 to 100% of the area examined. The following criteria were considered in this score: inflammation (edema, hemorrhage, and presence of inflammatory cells) and changes in muscle fibers (loss of striations, loss of cytoplasm, vacuolation, swelling, and hypercontraction bands).

### *C. perfringens* immunohistochemistry.

Sections of skeletal muscle were processed by an indirect immunoperoxidase technique for C. perfringens as previously described (44) using a Dako EnVision kit (Dako, Carpinteria, CA) according to the instructions of the manufacturer. The primary antibody was rabbit polyclonal C. perfringens antibody (GenWay Bio, San Diego, CA). Samples of skeletal muscle from mice receiving no C. perfringens inoculation were used as negative controls. Additional negative controls consisted of serial tissue sections of the test tissue incubated with normal rabbit serum instead of the specific antibodies. The colon of a goat from which C. perfringens had been isolated was used as a positive control for C. perfringens immunohistochemistry.

### Recovery of *C. perfringens* from challenged tissues.

Skeletal muscle was collected aseptically. Tissues were weighed, macerated, and resuspended in DPBS. Serial dilutions (10^−1^ to 10^−8^) in DPBS were plated on selective TSC agar plates for C. perfringens and anaerobically incubated overnight at 37°C. After 24 h, black colonies, indicative of C. perfringens (45), were counted to calculate the number of CFU per gram of muscle. A representative number (*n* = 15) of those colonies was screened by PCR for the *cpa* gene to confirm that they were C. perfringens. For this, we used the primers CPAF (5′-GCTAATGTTACTGCCGTTGA-3′) and CPAR (5′-CCTCTGATACATCGTGTAAG-3′), which amplify a PCR product of about 324 bp. The PCR program was as follows: initial denaturation at 94°C for 5 min; 30 cycles at 94°C for 45 s, 56°C for 45 s, and 72°C for 60 s; and final extension at 72°C for 7 min.

### Inhibition of gas gangrene by the 6-R peptide in a mouse model.

Aliquots (40 μl) of an overnight FTG cultures of wild-type, *agrB*-null mutant, or complementing strains were inoculated into 1 ml of TY medium containing 2 μl of the 6-R peptide (100 μM) in DMSO or an equal concentration of DMSO alone and then incubated at 37°C for 5 h. The cultures were then washed in sterile DPBS, and 1 μl of the 6-R peptide (100 μM) in DMSO or an equal concentration of DMSO alone was again added. An aliquot of 50 μl—equivalent to ∼10^6^ CFU—was injected intramuscularly in the left thighs of eight BALB/c mice (20 to 25 g) per treatment group. Another group of mice was injected with 50 μl of sterile DPBS (control). The animals were euthanized 4 h after infection, and samples of skeletal muscle were collected.

### Statistical analyses.

All statistical analyses were performed using R (v3.3.1). Histopathological scores were compared by using a nonparametric Kruskal-Wallis test, followed by the Dunn test as *post hoc* analysis. For CPA and PFO production in culture supernatants, one-way analysis of variance was applied followed by a *post hoc* analysis using Tukey’s multiple-comparison test. Bacterial counts were compared by negative binomial regression analysis. Differences were considered significant when the *P* value was <0.05.
